# Determining the Site of Upper Airway Narrowing in Snorers Using a Noninvasive Technique

**DOI:** 10.7759/cureus.28659

**Published:** 2022-09-01

**Authors:** Faris Alhejaili, Siraj O Wali, Shahd Abosoudah, Hani N Mufti, Hani Z Marzouki, Amir Ismail, Mohammed Abdelaziz, Ranya Alsumrani, Lama Rayyis, Elaf Alzarnougi, Jana Alkishi, Sarah Shaikhoon, Ghaedaa Alzahrani

**Affiliations:** 1 Sleep Medicine and Research Center, King Abdulaziz University Hospital, Jeddah, SAU; 2 Medical School, Royal College of Surgeons in Ireland, Ireland, IRL; 3 Medicine, King Abdullah International Medical Research Center, Jeddah, SAU; 4 Cardiac Surgery, King Faisal Cardiac Center, Jeddah, SAU; 5 Medicine, King Saud Bin Abdulaziz University for Health Sciences, Jeddah, SAU; 6 Otolaryngology - Head and Neck Surgery, King Abdulaziz University Hospital, Jeddah, SAU; 7 ENT Department, Tameside General Hospital, Ashton-Under-Lyne, GBR; 8 Neurology, King Faisal Specialist Hospital & Research Centre, Jeddah, SAU; 9 Internal Medicine, King Faisal Specialist Hospital & Research Centre, Jeddah, SAU; 10 Internal Medicine, King Abdulaziz University Hospital, Jeddah, SAU; 11 Endocrinology, King Abdulaziz University Hospital, Jeddah, SAU; 12 Radiation Oncology, King Fahad Medical City, Riyadh, SAU

**Keywords:** noninvasive technique, somnoscreen, nasopharyngoscopy, level of obstruction, snoring

## Abstract

Background

In this study, we aimed to determine the site of obstruction if surgical treatment is considered. Flexible nasopharyngoscopy is an invasive procedure currently used for the assessment of snoring and the level of obstruction. Here, we examine the role of Somnoscreen™ plus, a noninvasive cardiorespiratory polysomnographic device, in identifying the site of obstruction in patients presenting with snoring.

Methodology

This cross-sectional study was conducted in the Sleep Research Center at King Abdulaziz University Hospital. Polysomnography was conducted using Somnoscreen™ plus. All participants underwent flexible nasopharyngoscopy after polysomnography.

Results

Nasopharyngoscopy revealed that the most common site of obstruction was the nose and the soft palate (35.4%), followed by the soft palate alone (25%). Somnoscreen revealed that the site of obstruction was the nose and the soft palate in 18 (37.5%) patients and the nose alone in 16 (33.3%) patients. However, distal obstructions were not detected using Somnoscreen. The concordance of nasopharyngoscopy and Somnoscreen was 52.9%. However, it showed a discrepancy in identifying distal obstructions, which Somnoscreen™ plus failed to detect.

Conclusions

Somnoscreen appears to be sensitive for identifying proximal airway obstructions. The audio signal recordings can potentially be used as a tool to detect the site of airway obstruction in snoring; however, further studies are needed.

## Introduction

Snoring is a highly prevalent problem evident in approximately 20-40% of the general population [1]. A “snore” is the sound produced, mainly with inspiration during sleep, by the vibration of soft tissues in the upper airway because of a suboptimal airway muscle tone and/or an overpowering external force acting on the airway, causing it to collapse and restrict airflow [2]. This collapse may be partial or complete and has been implicated in obstructive sleep apnea (OSA) [1]. The more commonly affected sites are the soft palate, the base of the tongue, the epiglottis, and the pharynx [1]. While simple snoring is not proven to be associated with serious health complications, it can be a source of social nuisance and discomfort [2]. In addition, it is a key symptom linked with OSA; therefore, it may be an indicator of upper airway irregularity [3].

The third edition of the International Classification of Sleep Disorders (ICSD-3) defines OSA as a polysomnographically determined obstructive respiratory disturbance index (RDI) of ≥five events/hour associated with the typical symptoms of OSA (i.e., no refreshing sleep, daytime sleepiness, fatigue or insomnia, awakening with a gasping or choking sensation, loud snoring, or witnessed episodes of apnea) or an obstructive RDI of ≥15 events/hour, even in the absence of symptoms (ICSD-3). Nearly one billion individuals have mild-to-severe OSA worldwide [4]. Left untreated, OSA is known to contribute to daytime somnolence, intermittent hypoxia, weakened cognitive function, increased risk of carotid atherosclerosis, and stroke [1,5,6]. Consequently, this disease may have a negative impact on patients’ quality of life and safety [5]. Importantly, appropriate treatment interventions rely on accurate assessment of the site and degree of obstruction [7].

Among the available diagnostic tools used for assessing the upper airways are flexible nasopharyngoscopy and drug-induced sleep endoscopy (DISE). Flexible nasopharyngoscopy is an invasive endoscopic procedure used to visually examine the upper airway [7]. Although research indicates that it is a reliable tool for assessment, it requires training, is costly, and is associated with complications [1,8,9]. These include acute epiglottitis, laryngospasm, and bleeding [9]. DISE is a medical procedure that uses sedation to induce a sleep-like state and fiberoptic endoscopy to visualize the airway [10]. Nevertheless, a major disadvantage is that the snoring sounds produced during flexible nasopharyngoscopy do not precisely represent those of natural sleep [11]. In recent years, evidence has emerged supporting the use of acoustics to analyze snoring [1,12]. These techniques provide useful information for localizing the site of obstruction and may obviate the need for more invasive testing.

Concurrently, Somnoscreen™ plus is a noninvasive cardiorespiratory polysomnographic device with an inbuilt, high-sampling microphone that allows the recording of snoring sounds [13]. At present, research on the use of the Somnoscreen™ plus machine in determining the site of narrowing is not substantiated. Hence, this study aimed to validate its use compared with standard invasive methods.

## Materials and methods

Patients and methods

This was a cross-sectional study conducted in the Sleep Research Center at King Abdulaziz University Hospital in Jeddah between February 2017 and December 2019. The study was approved by the Research Ethics Committee at King Abdulaziz University Hospital (148-17). Participants older than 18, at risk of OSA as determined by the STOP-BANG and Epworth Sleepiness Scale, and complaining of snoring were included. Participation was voluntary and informed consent was obtained prior to enrollment. Participant demographic and polysomnographic data were collected. Following that, all participants underwent flexible nasopharyngoscopy by a certified ENT specialist.

Instruments

The STOP-BANG questionnaire was used to assess the risk of OSA. It is a clinical screening tool in which patients are given a set of eight yes or no questions that gather information on the following: (1) snoring, (2) tiredness, (3) observed apneas, (4) blood pressure, (5) body mass index (BMI), (6) age, (7) neck circumference, and (8) sex [14]. A score of 5 and above indicates a high risk of OSA, 3-4 indicates an intermediate risk, and 0-2 indicates low risk [14].

The Epworth Sleepiness Scale is a questionnaire that evaluates the extent of daytime sleepiness in patients at risk of OSA [15]. It involves eight questions about various daily situations in which patients may find themselves dozing off. Patients would then self-report using a scale from 0 to 3 of the likelihood that they might doze off in these different situations [15].

Polysomnography

Polysomnography is the gold standard study for OSA and involves monitoring patients using a wide range of detectors and sensors while sleeping [1,5,16]. In this study, polysomnography was conducted using Somnoscreen™ plus. It is a compact in-lab polysomnography system that consists of 17 channel head-box. It offers maximum data with high flexibility in providing sleep architecture and diagnosis. Somnoscreen™ plus device used for this study utilizes a high-sampling microphone to record patients’ snoring [13]. The high-sampling specialized microphone can record frequencies up to 4 kHz and is a useful tool to record sounds during sleep. During the polysomnography of the patient, the snoring noise is recorded. In addition to artificial noises, such as breathing and coughing, being reduced by the threshold value, the high-sampling specialized microphone is capable of filtering and achieving manual artifact control. The course of the evaluation of the snoring noise analysis is for nasal snoring, the base of the tongue, and the soft palate (velum) [13]. Topographic mapping is used to differentiate the sites of snoring depending on frequency analysis. Given that nasal snoring is 50-500 Hz, soft palate frequency is 500-800 Hz, and the root of the tongue ranges from 800 to 1,300 Hz. It is important to mention that whenever the term Somnoscreen™ plus device is used in this article we mean the device with a built-in high-sampling specialized microphone.

Flexible nasopharyngoscopy

A certified ENT specialist performed flexible nasopharyngoscopy for all patients while awake within one week of polysomnography in all participants. Flexible nasopharyngoscopy is an invasive endoscopic procedure for visual examination of the upper airway [7]. By visualizing the upper airway, localization of the site of obstruction would be possible and may enable the determination of the targeted therapy for OSA patients.

Statistical analysis

The median and interquartile range were used for continuous variables that were not normally distributed. To compare continuous variables, we applied the Wilcoxon rank-sum test. Categorical variables were reported using frequencies and percentages. Fisher’s exact test was used to evaluate the associations between categorical variables. Ordinal variables were analyzed using the Kruskal-Wallis test. All statistical tests were two-tailed, and p-values of <0.05 were considered significant.

Statistical analyses were performed using R software, version 4.0.2 (R Foundation for Statistical Computing, Vienna, Austria).

## Results

The demographic characteristics of the patient cohort are summarized in Table 1. A total of 48 patients were enrolled in the study. The median age and BMI were 46 years (IQR = 41-60) and 35.5 kg/m^2^ (IQR = 29.2-46), respectively, and 75% of the sample was male. Most patients were at high risk for OSA based on the STOP-BANG score (77%) (Table 1).

**Table 1:** Demographic characteristics of the patient cohort enrolled in the study. IQR: interquartile range; BMI: body mass index; ESS: Epworth Sleepiness Scale

Variable	N (%)	Median (IQR)
Age (years)		46 (41–60)
Male gender	36 (75)	
BMI (kg/m^2^)		35.5 (29.2–46)
ESS		12 (9–16)
STOP-BANG category		
Low risk	4 (8.3)	
Intermediate risk	5 (10.4)	
High risk	37 (77.1)	

All patients underwent polysomnography. The median total sleep time was 247 minutes, with a sleep efficacy of 65% and a median of 11 minutes in REM. Most patients spent approximately 57% of their time in a nonsupine position during the sleep study. The median respiratory distribution index was 15.1 (Table 2).

**Table 2:** Polysomnography variables and data. TST: total sleep time; AHI: apnea-hypopnea index; RDI: respiratory disturbance index

Variable	Median (IQR)
TST (minutes)	247 (186–290)
Sleep efficiency	65.2 (53.2–77.2)
REM TST (minutes)	10.8 (5.7–15.3)
N1 duration (minutes)	32 (20–49)
N2 duration (minutes)	117 (77–154)
N3 duration (minutes)	51 (27–71)
AHI	14.8 (8.4–31.4)
Sleep time (fraction)	
Supine	40.8 (13.4–81.8)
Not supine	57.6 (16.7–82)
RDI while supine	15.1 (3.5–41.2)

The locations of obstruction using flexible nasopharyngoscopy are summarized in Table 3. The ENT team examined all patients. The median neck circumference was 16.6 cm, and the Mallampati score was 3. The most common site of obstruction was the nose with the soft palate (35.4%), followed by the soft palate alone (25%) (Table 3). Although the location of snoring in the nose alone was detected in only eight (16.7%) patients, it seemed that the nose contributed to snoring in 33 (68.8%) patients as an isolated site or in combination with other locations. Somnoscreen™ plus revealed the site of obstruction to be at both the nose and the soft palate in 18 (37.5%) patients and at the nose alone in 16 (33.3%) patients. The nose was involved, separately or in combination with other sites, in 34 (70.8%) patients. Distal obstructions were not appreciated using this modality (Table 4).

**Table 3:** Location of the obstruction based on ENT examination using flexible nasopharyngoscopy.

Site of obstruction	N (%)
All three	3 (6.3)
Nose	8 (16.7)
Soft palate	12 (25)
Nose + soft palate	17 (35.4)
Nose + base of the tongue	5 (10.4)
Soft palate + base of the tongue	3 (6.3)

**Table 4:** Location of the obstruction based on Somnoscreen™.

Variable	N (%)
Site of obstruction	
Nose	16 (33.3)
Nose and soft palate	18 (37.5)
Not detected	14 (29.2)

**Figure 1:**
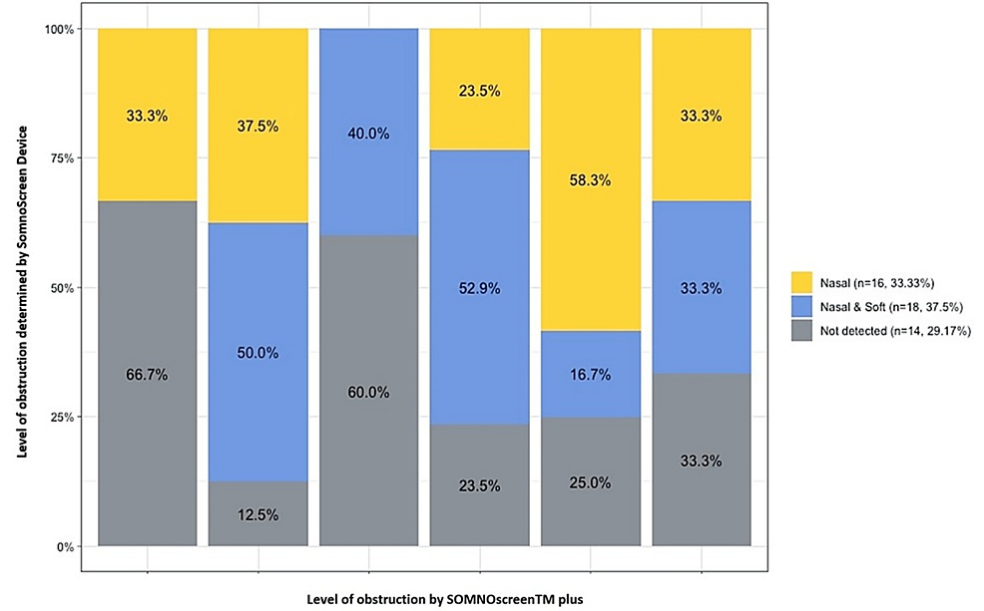
Comparison of Somnoscreen™ plus and flexible nasopharyngoscopy.

Figure 1 illustrates the concordance between Somnoscreen™ plus and flexible nasopharyngoscopy. The concordance of Somnoscreen™ plus and ENT examination was higher with nasal obstruction, but the noninvasive technique revealed additional obstruction caused by the soft palate, which was not detected by ENT nasopharyngoscopy. Meanwhile, 12.5% of patients found to have nasal obstruction by ENT nasopharyngoscopy were not detected by Somnoscreen™ plus.

When using Somnoscreen™ plus to examine patients found by flexible nasopharyngoscopy to have both nasal and soft palate obstruction, the concordance was 52.9%. In the remaining patients, Somnoscreen™ plus determined that the site of obstruction was only nasal (23.5%) or failed to detect any obstruction (23.5%). However, when the base of the tongue was implicated as a site of obstruction by ENT examination, the concordance of both methods was poor.

## Discussion

Snoring is a highly prevalent condition that indicates an irregularity in the upper airway that is evident during sleep and may indicate the presence of a serious underlying pathology such as OSA syndrome. Although polysomnography is the primary diagnostic tool for OSA, it does not identify the site of obstruction. In addition, not all patients who snore have OSA. Therefore, to successfully treat snoring, the site of narrowing needs to be determined. Although determining the site of obstruction in OSA may not be crucial when continuous positive airway pressure is used, this information may be very relevant when surgical treatment is contemplated. Likewise, finding the location of narrowing in OSA may influence the type of treatment, such as mandibular advancement devices. Flexible laryngoscopy offers a view of the entire upper airway, without the distortion resulting from anterior tongue retraction during the indirect examination. The anatomic findings dictate which type of tongue advancement/stabilization procedure is suggested, whether tongue reduction or lingual tonsillectomy is indicated, and whether epiglottis correction is needed.

Sometimes, it can be used under drug-induced sedation to assess for occult sites or patterns of obstruction and narrowing. Although it is variable, it has been shown to help predict surgical treatment success for some procedures such as assessing the state of the airway after completion of site-directed therapies and before maxillomandibular advancement [13]. In this study, we compared Somnoscreen™ plus with conventional flexible nasopharyngoscopy to determine the site of obstruction in patients with snoring.

In our study, flexible nasopharyngoscopy determined the site of obstruction to be the nose (16.7%), soft palate (25%), nose plus soft palate (35.5%), nose plus the base of the tongue (10.4%), and soft palate plus the base of the tongue (6.3%). Flexible nasopharyngoscopy can be performed while the patient is awake or anesthetized. In our study, the test was performed while the patient was awake. Although doing the test while the patient is awake can be a limitation for this test, and some may argue that it may not detect the true site of obstruction during sleep. Nevertheless, flexible nasopharyngoscopy may still give some idea about the likely site of obstruction. Studies by Cavaliere et al. comparing awake versus drug-induced sleep endoscopy showed that the pattern of obstruction caused by the soft palate and the tongue was similar in both techniques, but the latter tends to be more accurate with laryngeal involvement [17]. Therefore, Somnoscreen™ plus, being performed during sleep, may be superior to flexible nasopharyngoscopy in detecting the site of obstruction in OSA. We found that most of the narrowing was in the soft palate (oropharynx) with extension to the nasopharynx. Different studies have demonstrated variable results depending on the cohort of patients and the techniques used. The most common site of obstruction reported in these studies was at the level of the oropharynx, with extension to the laryngopharynx commonly observed [18]. Similarly, in a meta-analysis of articles that used DISE, the soft palate was obstructed in most patients with OSA, and the base of the tongue was obstructed in half of the patients [19].

We compared the results of Somnoscreen™ plus with the ENT physical examination. When the level of obstruction was at the nose on physical examination, the Somnoscreen™ plus device was 37.5% consistent. When the level of the obstruction was at the nose and the base of the tongue on physical examination, the Somnoscreen™ plus device was not able to detect any obstruction in 60% of patients, and 40% were labeled as having an obstruction at the nasal and soft palate level. The Somnoscreen™ plus device was most accurate when the level of obstruction on physical examination was at the nose and soft palate. The technique detected just over 50% of patients to have an obstruction at the level of the nose and soft palate.

The use of noninvasive methods, particularly acoustic frequencies, to evaluate the site of upper airway narrowing has recently attracted much interest. Pevernagie et al. [1] evaluated the acoustic frequencies of snoring using methods involved in speech analysis. The results of that study are promising and show that snoring sounds differ in frequencies depending on the site that is vibrating [1]. For example, tongue-base snoring tends to produce sounds of a higher frequency than palatal snoring [1]. Therefore, methods and devices, such as Somnoscreen™ plus, that are used to gather and analyze snoring sounds can localize sites of obstruction with minimal intervention and guide physicians toward the best interventions.

When assessing the concordance between Somnoscreen™ plus and ENT nasopharyngoscopy, we found discrepancies, especially with distal sites of obstruction. Somnoscreen™ plus failed to locate the base of the tongue as a site of obstruction or a source of snoring. However, the concordance of Somnoscreen™ plus with ENT examination was higher with proximal airway obstruction. Our study demonstrated that nasal obstruction detected by ENT examination was also detected in most cases by Somnoscreen™ plus. Interestingly, the noninvasive technique showed that there may have been additional obstruction caused by the soft palate that was not detected by ENT nasopharyngoscopy. In addition, 12.5% of patients labeled as having nasal obstruction by ENT nasopharyngoscopy were not detected by Somnoscreen™ plus.

When using Somnoscreen™ plus to examine patients found by flexible nasopharyngoscopy to have both nasal and soft palate obstruction, the concordance was 52.9%. In the remaining patients, Somnoscreen™ plus determined that the site of obstruction was only nasal (23.5%) or failed to detect any obstruction (23.5%). However, when the base of the tongue was implicated as a site of obstruction by ENT examination, the concordance of both methods was poor. Thus, it appears that the concordance of Somnoscreen™ plus with ENT examination is much higher with proximal airway obstruction than with distal airway obstruction.

Limitations of our study include its small sample size and the fact that both evaluations were operator-dependent. Even DISE is an operator-dependent test and has not been standardized. In fact, a study by Dijemeni et al. [20] found no universally accepted DISE classification system for analyzing the level and severity of anatomical or structural obstruction. While some clinicians preferred to use anatomical levels, others preferred anatomical structures. Furthermore, there is no agreement on describing the degree of obstruction detected by flexible nasopharyngoscopy. Additionally, in DISE studies, cardiopulmonary parameters were not recorded. It was observed that there is no robust DISE-classified treatment-planning framework for selecting appropriate surgical treatment for OSA. Similarly, there is no objective assessment of treatment results following flexible nasopharyngoscopy assessment [20]. Therefore, more studies with robust methodology are needed to assess the site of obstruction.

## Conclusions

The Somnoscreen™ plus device did not perform well when the level of airway obstruction was distal to the base of the tongue. It seems that this modality is sensitive to proximal rather than distal airway obstruction. Our study suggests that audio signal recordings have the potential to be used as a new and exciting tool to identify the predominant location of airway obstruction. However, more studies are needed to substantiate and evaluate the accuracy of this technique.
